# Leishmanicidal and Immunomodulatory Activities of the Palladacycle Complex DPPE 1.1, a Potential Candidate for Treatment of Cutaneous Leishmaniasis

**DOI:** 10.3389/fmicb.2018.01427

**Published:** 2018-07-03

**Authors:** Isabela B. dos Santos, Danielle A. M. da Silva, Fabiana A. C. R. Paz, Daniel M. Garcia, Adriana K. Carmona, Daniela Teixeira, Ieda M. Longo-Maugéri, Simone Katz, Clara L. Barbiéri

**Affiliations:** ^1^Departamento de Microbiologia, Imunologia e Parasitologia, Escola Paulista de Medicina, Universidade Federal de São Paulo, São Paulo, Brazil; ^2^Departamento de Farmacologia, Escola Paulista de Medicina, Universidade Federal de São Paulo, São Paulo, Brazil; ^3^Departamento de Biofísica, Escola Paulista de Medicina, Universidade Federal de São Paulo, São Paulo, Brazil

**Keywords:** cutaneous leishmaniasis, leishmaniasis treatment, *Leishmania (Leishmania) amazonensis*, palladacycle complex, immunomodulation

## Abstract

The present study focused on the activity of the palladacycle complex DPPE 1.1 on *Leishmania (Leishmania) amazonensis*. Promastigotes of *L. (L.) amazonensis* were destroyed *in vitro* by nanomolar concentrations of DPPE 1.1, whereas intracellular amastigotes were killed at drug concentrations fivefold less toxic than those harmful to macrophages. *L. (L.) amazonensis*-infected BALB/c mice were treated by intralesional injection of DPPE 1.1. Animals treated with 3.5 and 7.0 mg/kg of DPPE 1.1 showed a significant decrease of foot lesion sizes and a parasite load reduction of 93 and 99%, respectively, when compared to untreated controls. Furthermore, DPPE 1.1 was non-toxic to treated animals. The cathepsin B activity of *L. (L.) amazonensis* amastigotes was inhibited by DPPE 1.1 as demonstrated spectrofluorometrically by use of a specific fluorogenic substrate. Analysis of T-cells populations in mice treated with DPPE 1.1 and untreated controls was performed by fluorescence-activated cell sorter (FACS). IFN-γ was measured in supernatants of lymphocytes from popliteal and inguinal lymph nodes isolated from treated and untreated mice and stimulated with *L. (L.) amazonensis amastigotes* extract and active TGF-β was evaluated in supernatants of foot lesions; both dosages were carried out by means of a double-sandwich ELISA assay. A significant increase of TCD4^+^ and TCD8^+^ lymphocytes and IFN-γ secretion was displayed in mice treated with DPPE 1.1 compared to untreated animals, whereas a significant reduction of active TGF-β was observed in treated mice. These findings open perspectives for further investment in DPPE 1.1 as an alternative option for the chemotherapy of cutaneous leishmaniasis.

## Introduction

Leishmaniasis comprise a group of parasitic diseases displaying a wide clinical spectrum ranging from cutaneous, mucocutaneous, and visceral leishmaniasis. The World Health Organization reports a worldwide annual incidence of 0.6–1.0 million of new cases of cutaneous leishmaniasis and 50,000–90,000 of visceral leishmaniasis and 20,000–30,000 deaths occur annually (WHO, 2017). In the Amazon region, Brazil, *Leishmania (Leishmania) amazonensis* is one of the causative agents of human cutaneous leishmaniasis implicated with both the simple and diffuse forms of the disease (Lainson and Shaw, 1998). The drugs of choice for the treatment of these diseases are pentavalent antimonials, whereas amphotericin B and pentamidine represent the second-line therapy. However, toxicity, parasite resistance, high price, long treatment regimen, and mode of administration have limited the use of these compounds (Alvar et al., 2006; Polonio and Efferth, 2008; Goto and Lindoso, 2010). Other alternatives for treatment of leishmaniasis are miltefosine, paromomycin, and sitamaquine. Although these compounds have shown efficacy against cutaneous and visceral leishmaniasis they have restricted use due to host teratogenicity, development of parasite resistance and induction of undesirable adverse effects (Thakur et al., 2000; Sundar et al., 2002; Croft and Coombs, 2003; Soto et al., 2004; Jha et al., 2005). Therefore, the development of new leishmanicidal drugs is a priority for the control of leishmaniasis and has led to the development of new synthetic and natural products extracted from plants and marine sources which have displayed different degrees of efficacy in the treatment of experimental leishmaniasis (Sen and Chatterjee, 2011; Tempone et al., 2011). More recently, several findings suggest that compounds that activate the host immune system enhance the efficacy of antileishmanial drugs (Gupta et al., 2011; Seifert et al., 2015). Antitumoral drugs have also exhibited antileishmanial activity, leading to the screening of these compounds *in vitro* and in clinical trials (Fuertes et al., 2008; Sanderson et al., 2014). Among antitumoral drugs, cyclopalladated complexes have shown low toxicity in animals and some of them exhibited leishmanicidal and tripanocidal activity (Caires, 2007; Navarro et al., 2008; Matsuo et al., 2010; Velásquez et al., 2016). Furthermore, inhibition of cathepsin B activity has been implicated in destruction of tumoral cells by palladacycle complexes and their inhibitory effect on *Leishmania* cysteine proteases *in vitro* was also demonstrated (Bincoletto et al., 2005; Fricker et al., 2008). More recently, the effect of the palladacycle complex DPPE 1.2 on *in vitro* and *in vivo* *L. (L.) amazonensis* infection was reported (Paladi et al., 2012). The present study shows the action of the palladacycle complex DPPE 1.1 on promastigotes, intracellular amastigotes, and cutaneous lesions in BALB/c mice infected with *L. (L.) amazonensis*. Furthermore, the high efficacy of DPPE 1.1 on *L. (L.) amazonensis* infection *in vivo* is followed by the modulation of the host immune responses.

## Materials and Methods

### Animals

Eight-week-old female Golden hamsters were obtained from breeding stocks of Anilab Company, Paulínia (São Paulo, Brazil). Female BALB/c mice 6–8 weeks old were acquired from Universidade Federal de São Paulo (São Paulo, Brazil). All animals were bred and housed under specific pathogen-free conditions and fed a regular diet. All animal procedures were carried out in strict accordance with the recommendations in the Guide for the Care and Use of Laboratory Animals of the Brazilian National Council of Animal Experimentation^1^. The protocol was approved by the Committee on the Ethics of Animal Experiments of the Institutional Animal Care and Use Committee at the Federal University of São Paulo (Id # CEUA 127520).

### Parasites

The *L. (L.) amazonensis* strain used (MHOM/BR/1973/M2269) was kindly provided by Dr. Jeffrey J. Shaw, Instituto Evandro Chagas, Belém, Pará, Brazil and maintained as amastigotes by inoculation into footpads of Golden hamsters every 4–6 weeks as previously described (Barbiéri et al., 1990). *L. (L.) amazonensis* promastigotes were grown at 26°C in 199 medium (Gibco) supplemented with 4.2 mM sodium bicarbonate, 4.2 mM HEPES, 1 mM adenine, 5 mg/ml hemin (bovine type I) (Sigma-Aldrich, St. Louis, MO, United States), 100 U/ml penicillin, 100 μg/ml streptomycin, and 10% fetal calf serum (FCS) (Cultilab, SP, Brazil).

### Biphosphinic Palladacycle Complex [Pd_2_(S(-)C_2_, N-DMPA)_2_(m-DPPE)]Cl_2_(DPPE 1.1)

**FIGURE 1:**
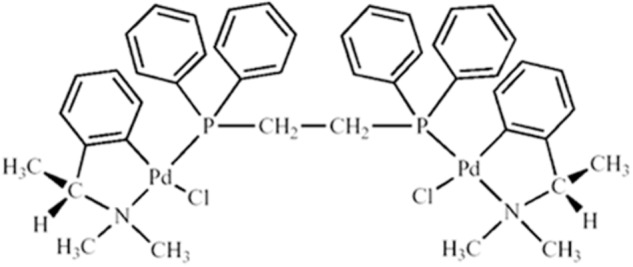
Structure of the DPPE 1.1 compound [Pd_2_(S(-)C_2_,N-DMPA)_2_ (m-DPPE)]Cl_2_.

The palladacycle compound DPPE 1.1 (**Figure 1**) was obtained from N,N-dimethyl-1-phenethylamine (DMPA), complexed to 1,2-bis(diphenylphosphino)ethane (DPPE) ligand and synthesized as previously described (Rodrigues et al., 2003). Stock solutions at 1.0 mM were prepared in PBS after solubilization in dimethylsulfoxide (final concentration of 0.1%). For *in vitro* experiments, the drug was diluted to the appropriate concentration in cell culture medium, and for *in vivo* injections the stock was diluted in PBS.

### Effect of DPPE 1.1 on *L. (L.) amazonensis* Promastigotes and Intracellular Amastigotes

The promastigote cultures at 1 × 10^6^ parasites/ml were kept in 199 culture medium as described above containing between 2.5 and 50 nM of DPPE 1.1. Parasites were counted daily in a Neubauer chamber for 3 days. The leishmanicidal effect of DPPE 1.1 on intracellular amastigotes was evaluated in mouse bone marrow derived macrophages infected with *L. (L.) amazonensis*. Bone marrow-derived macrophages were generated from bone marrow stem cells isolated from BALB/c mice (Zamboni and Rabinovitch, 2003). Cells were counted, added (8 × 10^5^) and cultured on glass coverslips inserted in 24-well tissue culture plates containing RPMI 1640 medium buffered with 15 mM of HEPES, 20 mM of sodium bicarbonate and supplemented with 1 mM L-glutamine, 20% of FCS, 100 U/ml penicillin, 100 μg/ml streptomycin, and 30% L929 cell conditioned medium. Cultures were kept at 37°C in an atmosphere of air/CO_2_ (95/5%). After 5 days, the medium was changed for RPMI containing 10% of FCS and macrophages were infected at a multiplicity of 2 amastigotes per macrophage. After 24 h, infected cultures were treated with different drug concentrations (125–750 nM) for 3 days. The coverslips were fixed with methanol, stained with hematoxylin-eosin and intracellular amastigotes were counted. Results are expressed by the infection index, obtained by multiplying the percentage of infected macrophages by the average number of amastigotes per macrophage. At least 200 macrophages were scored in each three coverslips. Amphotericin B at 200 nM (Sigma-Aldrich, St. Louis, MO, United States) and 1.4 mg/ml (approximately 2.76 mM) of Glucantime (Sanofi-Aventis, Brazil, 300 mg/ml, 81 mg/ml SbV) were used as standard drugs for treatment of *L. (L.) amazonensis* promastigotes and intracellular amastigotes, respectively.

### Inhibition of *L. (L.) amazonensis* Cathepsin B by DPPE 1.1

Cathepsin activity was monitored with the fluorogenic substrate Abz-Gly-Ile-Val-Arg-Ala-Lys(Dnp)-OH (Sigma, St. Louis, MO, United States), specific for cathepsin B (Cotrin et al., 2004), using 2 μg of *L. (L.) amazonensis* amastigote cell lysate (1 × 10^9^ amastigotes disrupted in 200 μl PBS), 1 ml of 100 mM acetic acid, pH 4.5, containing 200 mM of NaCl, 10 μg of substrate in the presence of either increasing concentrations of DPPE 1.1 or CA074, a specific inhibitor of cathepsin B. The cathepsin activity was monitored spectrofluorometrically using the fluorogenic substrate on a Hitachi F-2000 spectrofluorometer equipped with a thermostated cell holder. The fluorescence excitation (λEx) and emission (λEm) wavelengths for the fluorescence of Abz-peptide fragments resulting from the Abz-Gly-Ile-Val-Arg-Ala-Lys(Dnp)-OH hydrolysis were set at 320 and 420 nm, respectively.

### *In Vivo* Antileishmanial Assays

*In vivo* leishmanicidal activity of DPPE 1.1 was evaluated in female BALB/c mice 6–8 weeks-old infected subcutaneously at the right hind-foot with 1 × 10^5^ *L. (L.) amazonensis* amastigotes freshly prepared as previously described. Ten days after infection, the animals were randomly separated in two groups of 12 mice each. Treated animals received in the foot lesions every other day doses of 235 μg/kg/day of DPPE 1.1 for 5 and 10 weeks (total of 3.5/Kg and 7.0 mg/Kg, respectively). Control group received the same number of injections of PBS. Infection was monitored once a week by measuring the diameter of foot lesions with a dial caliper (Mitutoyo Corporation, Japan). The animals were euthanized 7 days after the end of each period of treatment. Parasite burden was determined by limiting dilution in foot lesions, as previously described (Lima et al., 1997) and lymphocytes were isolated from popliteal and inguinal lymph nodes for evaluation of immune responses.

### Assays for Toxicity

Serum concentrations of urea, creatinine, and transaminases were determined in BALB/c mice 7 days after the end of treatment for 10 weeks, using sets of commercial reagents (Doles Reagentes e Equipamentos para Laboratórios Ltda, Brazil).

### Evaluation of Immune Responses

The T lymphocyte population was analyzed by FACS (Afar et al., 1991). After isolation from popliteal and inguinal lymph nodes and washing with PBS, 1 × 10^6^ lymphocytes were fixed in formalin 1% in PBS for 30 min at 4°C, washed twice in PBS, resuspended in PBS and incubated with monoclonal antibodies either anti-CD3 conjugated to allophycocyanin, or anti-CD4 conjugated to phycoerythrin or anti-CD8 conjugated to peridinin chlorophyll protein (Pharmingen). After 1 h at 4°C, they were washed twice in PBS, fixed in formalin 1% in PBS for 30 min at 4°C, washed twice in PBS, resuspended in PBS and gated on the basis of forward-angle and right-angle scatter and the fluorescence intensity was analyzed by FACS (FACSCAN—Cell Sorter Becton–Dickinson).

For evaluation of IFN-γ production, 1 × 10^6^ lymphocytes isolated from popliteal and inguinal lymph nodes were cultured in 200 μl of RPMI 1640 containing 20 mM NaHCO_3_, 10 mM Hepes, 100 U/ml penicillin, 100 μg/ml streptomycin, 2 mM L-glutamine, 50 μM β-mercaptoethanol, 5 mM sodium pyruvate, 100 μM of non-essential amino acids solution and 10% FCS and maintained for 72 h in the presence of 50 μg/ml of *L. (L.) amazonensis* amastigote extract. Supernatants from lymphocytes cultured in the absence of parasite extract were used as a negative control. Culture supernatants were tested for IFN-γ using a double-sandwich enzyme-linked immunosorbent assay according to the manufacturer instructions (eBioscience, Inc., San Diego, CA, United States). Detection of active TGF-β was carried out in the supernatants of foot lesions from BALB/c mice isolated 7 days after the end of treatment for 5 weeks with DPPE 1.1. The animals were euthanized, and after homogenization of excised lesions in PBS, supernatants were collected, cleared by centrifugation and assayed for TGF-β by a double-sandwich ELISA assay (eBioscience, Inc., San Diego, CA, United States). Supernatant concentrations higher than the minimal values obtained from the IFN-γ and TGF-β standard were considered to be positive.

### Statistical Analysis

One-way ANOVA and Student’s *t*-test were used to determine the significant differences between groups by use of GraphPad Prism (version 5.0) and *P*-values smaller than 0.05 (*P* < 0.05) were considered significant.

## Results

### Growth Inhibition of *L. (L.) amazonensis* Promastigotes by DPPE 1.1

Growth of *L. (L.) amazonensis* promastigotes was significantly inhibited in the presence of 2.5 to 50 nM of DPPE 1.1 after 2 and 3 days of treatment. Nearly 100% of promastigotes were killed after 2, 3, and 4 days in the presence of 20 and 50 nM of DPPE 1.1. A growth curve similar to control was observed when *L. (L.) amazonensis* promastigotes were cultured in the presence of the highest concentration of DMSO used for DPPE 1.1 solubilization (0.04%). Parasites were also grown in the presence of amphotericin B and after 72 of incubation the IC_50_ and IC_90_ values for both drugs were determined (**Table 1**).

**Table 1:** *In vitro* activity of DPPE 1.1 and amphotericin B on *L. (L.) amazonensis* promastigotes.

Drug	IC_50_ (nM)	CI 95%	IC_90_
DPPE 1.1	2.72	2.649–2.8022	5.99
Amphotericin B	27.36	22.08–33.88	142.3

### Decrease of Infection in Macrophages Harboring *L. (L.) amazonensis* After Treatment With DPPE 1.1

**FIGURE 2:**
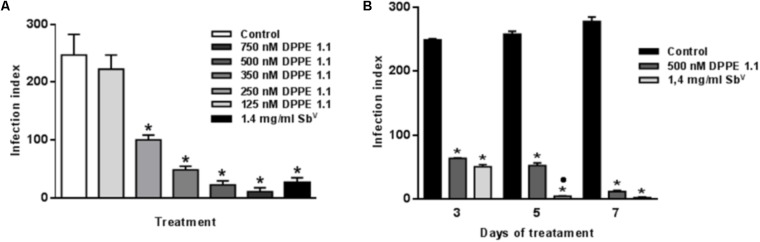
**(A)** Activity of DPPE 1.1 on *L. (L.) amazonensis*-infected macrophages. Mouse bone marrow derived macrophages infected with amastigotes of *L. (L.) amazonensis* were treated with the drugs for 3 days and the infection index was estimated. ^∗^*P* < 0.001 compared to control. **(B)** Kinetics of DPPE 1.1 leishmanicidal activity on *L. (L.) amazonensis*-infected macrophages. Mouse bone marrow derived macrophages were infected with amastigotes of *L. (L.) amazonensis*, treated with the drugs for 3, 5, and 7 days and the infection index was calculated after each period. ^∗^*P* < 0.001 compared to control; ^⋅^*P* < 0.05 compared to treatment with DPPE 1.1 500 nM.

Mouse bone marrow derived macrophages infected with *L. (L.) amazonensis* amastigotes for 24 h were treated with several concentrations of DPPE 1.1 ranging from 125 to 750 nM. Treatment of infected cultures with Glucantime 1.4 mg/ml was used as a positive control. Three days after incubation, infected macrophages were fixed and stained for parasite counts and determination of the infection index. A significant, dose-dependent decrease in macrophage infection was observed with an inhibition of 95% for 750 nM of DPPE 1.1 (IC_50_ of 227.7 nM; 95% confidence limits, 219.28–236.59 nM) (**Figure 2A**). Kinetics assays showed an increasing leishmanicidal effect of DPPE 1.1 at longer periods of treatment (**Figure 2B**). Infected cultures were also incubated with the highest concentration of DMSO used for DPPE 1.1 solubilization (0.04%) and no reduction of the viability or the infection of macrophages was observed (data not shown). The cytotoxicity of DPPE 1.1 on macrophages was evaluated by the 3-(4,5-dimethylthiazol-2-yl)-2,5-diphenyltetrazolium bromide (MTT) method and the CC_50_ was determined (1,236 nM; 95% confidence limits, 1.19–1.28 nM). Leishmanicidal effect on intracellular amastigotes was also observed in the presence of 1.4 mg/ml of Glucantime (approximately 2.76 mM) and treatment with this antimonial at concentrations higher than 1.4 mg/ml resulted in macrophage toxicity (data not shown).

### DPPE 1.1 Inhibits the Cathepsin B Activity of *L. (L.) amazonensis* Amastigotes

**FIGURE 3:**
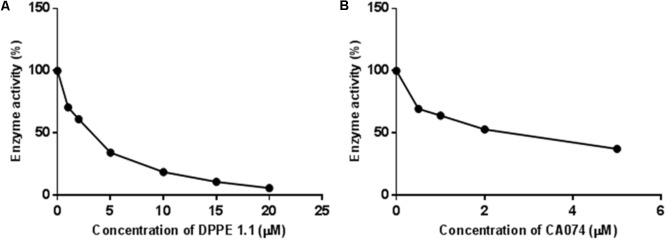
Effect of DPPE 1.1on proteolytic activity of *L. (L.) amazonensis*. Fluorogenic substrate specific for cathepsin B-like proteases was incubated with extracts of *L. (L.) amazonensis* amastigotes in presence of increasing concentrations of DPPE 1.1 **(A)** or CA074 **(B)**.

DPPE 1.1 or CA074 significantly inhibited the activity of *L. (L.) amazonensis* extract on a most specific substrate for cathepsin B (**Figure 3**). A previous spectrofluorometric assay with specific substrates for cathepsins was performed and showed that although DPPE 1.1 inhibited the enzymatic activity on all of them, a significantly higher reduction on cathepsin B activity could be observed in the presence of this palladacycle complex (data not shown). These data led us to test the activity of *L. (L.) amazonensis* extract on a most specific substrate for cathepsin B. **Figure 3** shows that the parasite proteolytic activity was significantly inhibited either by DPPE 1.1 or CA074. The calculated IC_50_ values for DPPE 1.1 and CA074 were not significantly different (4,413 and 4,540 μM, respectively), strongly suggesting that DPPE 1.1 inhibits *L. (L.) amazonensis* cathepsin B.

### Reduction of Parasite Load in *L. (L.) amazonensis*-Infected BALB/c Mice After Treatment With DPPE 1.1

**FIGURE 4:**
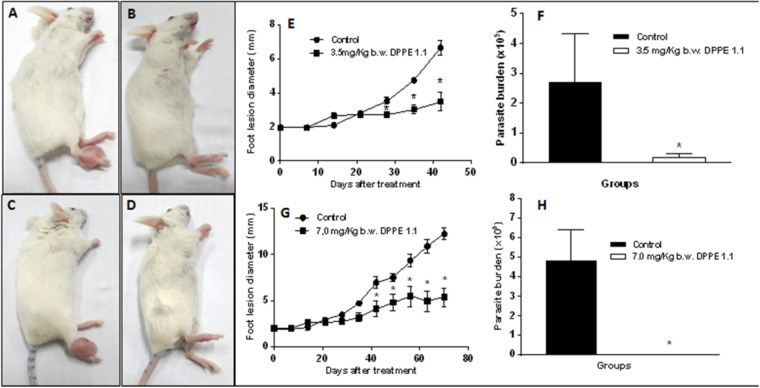
Effect of DPPE 1.1 on BALB/c mice infected with *L. (L.) amazonensis*. Macroscopic evaluation of lesions in untreated mice **(A,C)**, mice treated with DPPE 1.1 for 5 (3.5 mg/Kg) **(B)** and 10 weeks (7.0 mg/Kg) **(D)**. Development of foot lesions in *L. (L.) amazonensis*-infected BALB/c mice treated with DPPE 1.1 for 5 **(E)** and 10 weeks **(G)**. Parasite load in foot lesions of *L. (L.) amazonensis-*infected BALB/c mice treated with DPPE 1.1 for 5 **(F)** and 10 weeks **(H)**. ^∗^*P* < 0.05. Data are representative of three independent experiments.

BALB/c mice infected with *L. (L.) amazonensis* were treated every other day with 235 mg/kg/day of DPPE 1.1 for 5 and 10 weeks injected in foot lesions. As can be observed in **Figure 4**, starting from 28 days of treatment the animals which received DPPE 1.1 showed a significant decrease of foot lesion size compared to controls until the end of treatment (**Figures 4B,E**). Parasite load was also determined by limiting dilution in foot lesions of BALB/c mice 7 days after the end of treatment for 5 and 10 weeks. **Figure 4** shows that BALB/c mice treated with DPPE 1.1 displayed a significant reduction of parasite load compared to untreated animals in both periods evaluated (**Figures 4F,H**). To evaluate hepato and nephrotoxicity of DPPE 1.1 serum levels of transaminases, urea and creatinine were determined. No statistically significant alterations were detected between groups (**Table 2**).

**Table 2:** Serum concentrations of transaminases, urea, and creatinine in *L. (L.) amazonensis*-infected BALB/c mice 7 days after treatment with either PBS or DPPE 1.1 for 10 weeks.

Groups	Biochemical markers	Concentration	Reference values
PBS	AST	13.968 UI/L	12–42 UI/L
DPPE 1.1	AST	13.531 UI/L	
PBS	ALT	9.856 UI/L	8–42 UI/L
DPPE 1.1	ALT	12.863 UI/L	
PBS	Urea	18.98 mg/dL	15–40 mg/dL
DPPE 1.1	Urea	24.36 mg/dL	
PBS	Creatinine	0.96 mg/dL	0.5–1.1 mg/dL
DPPE 1.1	Creatinine	0.74 mg/dL	

### Increase of TCD4^+^, TCD8^+^ Lymphocytes and IFN-γ Secretion and Reduction of TGF-β in *L. (L.) amazonensis*-Infected-BALB/c Mice After Treatment With DPPE 1.1

**FIGURE 5:**
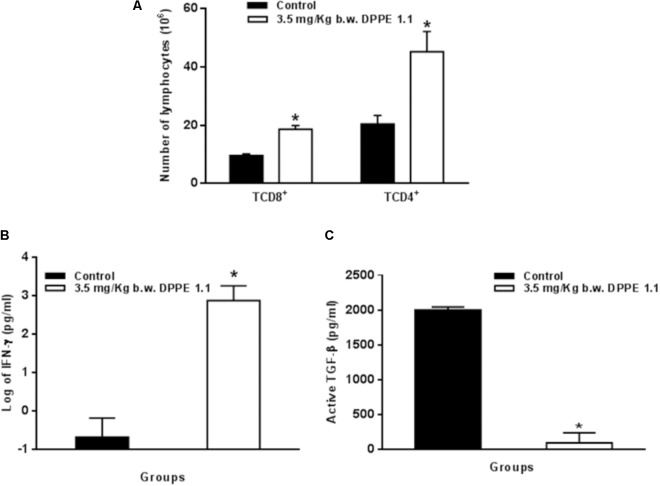
**(A)** Absolute numbers of T lymphocyte populations in treated mice. *L. (L.) amazonensis*-infected BALB/c mice were treated with DPPE 1.1. Lymphocytes were isolated from popliteal and inguinal lymph nodes, stained with monoclonal antibodies anti-CD3, anti-CD4, and anti-CD8 and analyzed by FACS. *^∗^P <* 0.05 compared to control that received PBS. **(B)** Secretion of IFN-γ in BALB/c mice treated with DPPE 1.1. Lymphocytes were isolated from popliteal and inguinal lymph nodes and after 72 h of stimulation *in vitro* with extract of *L. (L.) amazonensis* amastigotes IFN-γ was detected by ELISA assay in culture supernatants. **(C)** Evaluation of TGF-β in treated mice. Levels of active TGF-β were determined by ELISA assay in supernatants of foot lesions from *L. (L.) amazonensis* infected BALB/c after the treatment with DPPE 1.1. ^∗^*P* < 0.05 compared to control groups.

The analysis of T lymphocyte expression by FACS performed 5 weeks after the treatment with DPPE 1.1 showed a significant increase of TCD4^+^ and TCD8^+^ lymphocytes in BALB/c mice treated with DPPE 1.1 compared to control group (**Figure 5A** and **Supplementary Figure S1**). At the same period of treatment secretion of IFN-γ and TGF-β was also evaluated. A significant increase of IFN-γ secretion was observed in mice treated with DPPE 1.1 compared to untreated animals (**Figure 5B**). High levels of active TGF-β were detected in the foot lesions from mice that received PBS in contrast to those treated with DPPE 1.1 that displayed a significant reduction of TGF-β production (**Figure 5C**).

## Discussion

The present study showed the *in vitro* and *in vivo* activity of the palladacycle complex DPPE 1.1 on *L. (L.) amazonensis*. The *in vitro* leishmanicidal action of DPPE 1.1 was firstly demonstrated on *L. (L.) amazonensis* promastigotes which were destroyed at nanomolar concentrations of DPPE 1.1 with IC_50_ value 10-fold lower than that of Amphotericin B. This palladacycle complex also displayed an effective leishmanicidal activity on intracellular amastigotes (IC_50_ = 227.7 nM), while was fivefold less toxic to macrophages (CC_50_ = 1,236 nM). Similar leishmanicidal effect was also observed with Glucantime, but at significantly higher concentrations compared to those used with DPPE 1.1. The antiprotozoal action of DPPE 1.1 was previously demonstrated on *Trypanosoma cruzi* which was destroyed with lower concentrations of this palladacycle complex compared to benznidazole, the main drug available for the treatment of Chagas’ disease (Matsuo et al., 2010). Other palladacycles were tested against *Leishmania* and showed an effective activity only on promastigote growth (Fricker et al., 2008) and previous data from our group showed the *in vitro* and *in vivo* efficacy of the palladacycle complex DPPE 1.2 on *L. (L.) amazonensis* (Paladi et al., 2012). More recently the leishmanicidal activity of a series of palladacycles on intracellular amastigotes of *L. (L.) amazonensis* was also demonstrated and among them a binuclear palladacycle complex named CP2 showed an effective action on *L. (L.) amazonensis* infection (Velásquez et al., 2016, 2017). The leishmanicidal activity of DPPE 1.1 is comparable to that observed with CP2. However, a concentration 44-times higher of CP2 was used to destroy *L. (L.) amazonensis* amastigotes *in vitro* in comparison to DPPE 1.1 (Velásquez et al., 2017).

The leishmanicidal effect of DPPE 1.1 was also demonstrated *in vivo*. Treatment with 3.5 and 7 mg/kg of DPPE 1.1 led to a reduction of parasite load in foot lesions of 93 and 99%, respectively. In comparison with *in vivo* treatment with CP2, our data indicate the higher efficacy of DPPE 1.1 over this cyclopalladated compound since at the same concentration (7 mg/kg) mice treated with DPPE 1.1 and CP2 displayed a decrease of parasite load of 99 and 55%, respectively (Velásquez et al., 2017). Furthermore, treatment with DPPE 1.1 did not result in toxicity in *L. (L.) amazonensis*-infected BALB/c mice as demonstrated by hepatic and renal assays after treatment with this compound, corroborating literature data that showed the low toxicity of these palladacycle complexes in the treatment of mice against tumor cells (Rodrigues et al., 2003). Although the treatment with DPPE 1.1 resulted in a high reduction of parasite load in foot lesions (93 and 99%), there was no a sterile cure in treated mice. However, it is important to note that the BALB/c strain is highly susceptible to *L. (L.) amazonensis* infection and mimics the anergic form of diffuse cutaneous leishmaniasis caused by this parasite (Andrade et al., 1984; Lainson and Shaw, 1987).

The inhibitory activity of palladacycle complexes on the cysteine protease cathepsin B of tumoral cells was reported and this property has been related, at least in part, to the antitumoral activity of these compounds (Bincoletto et al., 2005). We showed that DPPE 1.1 inhibited the high cathepsin B activity expressed in *L. (L.) amazonensis* amastigotes. The inhibitory effect of the palladacycle complex DPPE 1.2 on *L. (L.) amazonensis* cathepsin B activity was also demonstrated (Paladi et al., 2012). Although the involvement of cathepsin L-like and cathepsin B-like in *Leishmania* growth and virulence has been demonstrated *in vitro* and *in vivo* (Mottram et al., 1996, 1998; Bart et al., 1997), the relevance of the cathepsin B activity inhibition for destruction of *L. (L.) amazonensis* by DPPE 1.1 in treated mice needs to be further explored. Other possible relevant targets which have been associated to the action of palladacycles against tumor cells are their effects on the lysosomal and mitochondrial permeabilization that can trigger apoptosis (Barbosa et al., 2006; Santana et al., 2009). The induction of *L. (L.) amazonensis* apoptosis by DPPE 1.1 has been investigated. Although some evidence indicated that DPPE 1.1 can exert an apoptosis-like death in *L. (L.) amazonensis* promastigotes, these data need further confirmation that is under investigation.

The treatment of *L. (L.) amazonensis*-infected BALB/c mice with DPPE 1.1 was followed by the activation of the immune system as shown by the increase of TCD4^+^ and TCD8^+^ lymphocytes and the higher frequency of lymphocytes producing IFN-γ in treated animals. The participation of the immune system was also demonstrated in *L. (L.) amazonensis*-infected mice treated with the palladacycle complex DPPE 1.2 (Paladi et al., 2017). TGF-β was significantly reduced in mice after the treatment with DPPE 1.1. TGF-β is an immunosuppressor cytokine known to exacerbate visceral and cutaneous leishmaniasis (Barral-Netto et al., 1992; Rodrigues et al., 1998; Wilson et al., 1998; Pinheiro et al., 2005). The present data corroborate these findings since low levels of TGF-β were detected in foot lesions from mice treated with DPPE 1.1 followed by a significant increase of CD4^+^ and CD8^+^ T lymphocytes and IFN-γ secretion. Literature data have also demonstrated that TGF-β is an important factor in the matrix formation and stimulation of collagen production, playing a key role in the acceleration of wound healing in cutaneous leishmaniasis (Abdoli et al., 2017). In the present study the collagen production was not analyzed. However, our data seem not support this hypothesis since in mice treated with DPPE 1.1 there was a significant decrease of TGF-β. Data on IFN-γ production suggest the participation of this cytokine in mice treated with DPPE 1.1. Although the involvement of CD4^+^ Th1 lymphocytes producing IFN-γ has been demonstrated in mice protected against *L. (L.) amazonensis* infection (Coelho et al., 2003; Campbell et al., 2004; Pinto et al., 2004), our previous data showed that the strain of *L. (L.) amazonensis* used in the present study is unresponsive to nitric oxide secreted by activated macrophages (Carmo et al., 2010). Furthermore, literature data have shown that it is controversial the susceptibility of *L. (L.) amazonensis* to nitric oxide secreted by activated macrophages (Qi et al., 2004; Mukbel et al., 2007). Therefore, it is possible that cytotoxic CD8^+^ lymphocytes play a more relevant role in destruction of *L. (L.) amazonensis* in mice treated with DPPE 1.1, corroborating previous data which showed the participation of cytotoxic CD8^+^ T lymphocytes in *L. (L.) amazonensis* infection (Colmenares et al., 2003; Alves et al., 2004; Pereira and Alves, 2008; Pereira et al., 2011; Souza-Silva et al., 2014). Furthermore, our previous data showed an increase of CD8^+^ T lymphocytes parallel to their cytotoxic activity on *L. (L.) amazonensis*-infected macrophages (Fedeli et al., 2010).

## Conclusion

In conclusion, the leishmanicidal and immunomodulatory activity of DPPE 1.1 against *L. (L.) amazonensis* infection at concentrations non-toxic to the host support further studies to explore the potential of this palladacycle complex as an additional option to available chemotherapies for leishmaniasis.

## Author Contributions

IS, DS, FP, AC, DT, and SK designed and performed the experiments, analyzed and interpreted the data. DG designed, synthesized, and analyzed the compound. IL-M analyzed and interpreted the data and contributed to reviewing of manuscript. CB conceived the work, contributed to interpretation of data, wrote and reviewed the manuscript.

## Conflict of Interest Statement

The authors declare that the research was conducted in the absence of any commercial or financial relationships that could be construed as a potential conflict of interest.

## Footnotes

1 http://www.sbcal.org.br

## Supplementary Material

FIGURE S1 Gate strategy of the T lymphocyte subsets from popliteal and inguinal lymph nodes obtained of control and group treated with DPPE 1.1. **(A)** Dot plot forward scatter (FSC) × side scatter (SSC) gated on lymphocyte population. **(B)** Dot plot gated on CD3^+^ cells. **(C)** Dot plot gated on CD4^+^ and CD8^+^.
