# Supplementary figures and images for: Leishmanicidal and Immunomodulatory Activities of the Palladacycle Complex DPPE 1.1, a Potential Candidate for Treatment of Cutaneous Leishmaniasis
Source: Front Microbiol. 2018 Jul 3;9:1427. doi: 10.3389/fmicb.2018.01427 (PMC6038773; doi:10.3389/fmicb.2018.01427)

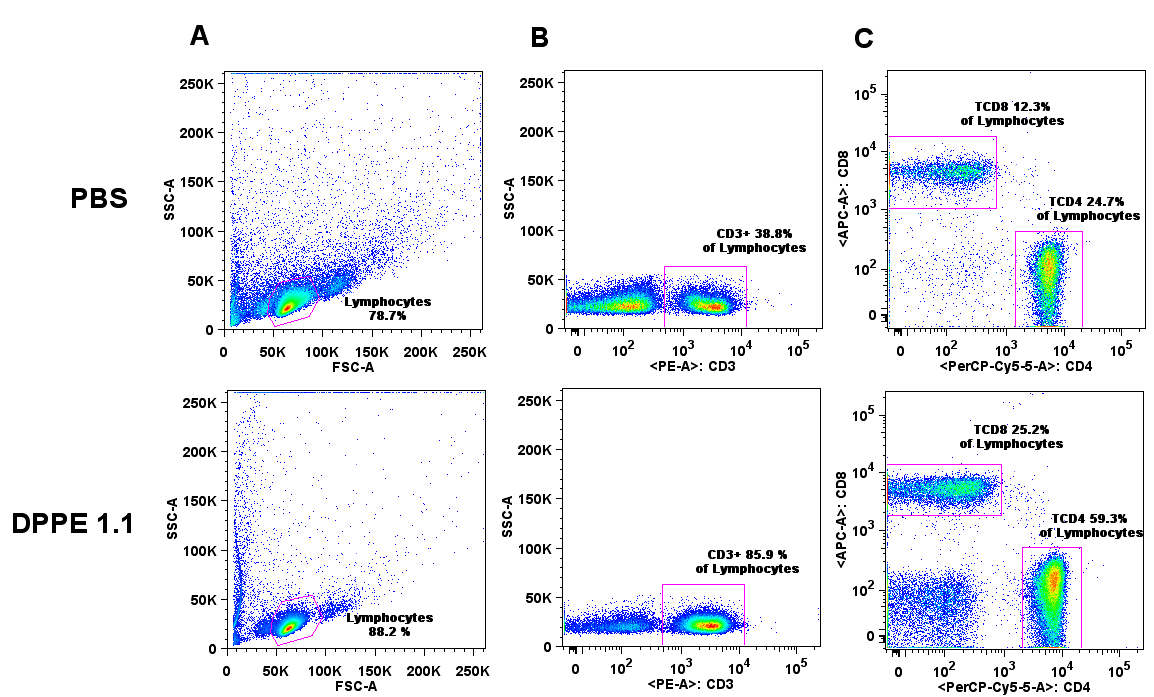

Supplement: FIGURE S1 — Gate strategy of the T lymphocyte subsets from popliteal and inguinal lymph nodes obtained of control and group treated with DPPE 1.1. (A) Dot plot forward scatter (FSC) × side scatter (SSC) gated on lymphocyte population. (B) Dot plot gated on CD3+ cells. (C) Dot plot gated on CD4+ and CD8+. [file Image_1.TIF]
